# The socioeconomic context of stigma: examining the relationship between economic conditions and attitudes towards people with mental illness across European countries

**DOI:** 10.3389/fepid.2023.1076188

**Published:** 2023-07-19

**Authors:** Katie Pybus, Kate E. Pickett, Charlie Lloyd, Richard Wilkinson

**Affiliations:** Department of Health Sciences, University of York, York, United Kingdom

**Keywords:** stigma, mental illness, socioeconomic, income inequality, public attitudes

## Abstract

**Introduction:**

Efforts to reduce the stigma associated with mental illness have intensified over the past 30 years with a particular focus on improving public attitudes. Difficult economic circumstances can be harmful to intergroup relations, but little is known about whether there is a relationship between socioeconomic conditions and attitudes towards people with mental illnesses.

**Methods:**

Random effects logistic regression modelling was employed to explore the relationship between individual financial circumstances, contextual socioeconomic factors and difficulty speaking to a person with a significant mental illness across European countries.

**Results:**

Lower GDP per capita and higher income inequality at the country level, alongside individual financial difficulties, were each associated with a greater likelihood of reporting difficulty speaking to a person with a significant mental illness.

**Discussion:**

Micro and macro-economic factors are associated with public attitudes towards people with mental illness across Europe. With prolonged economic instability predicted over the coming years in Europe it is important that these findings are taken into consideration when designing mental health and social policies, in order to safeguard the progress that has been made in reducing mental health stigma to date.

## 1. Background

The stigma associated with mental illness has wide-reaching and detrimental effects on those who experience it, including depression, low self-esteem and broader inequalities in life chances (1–3). The past 30 years has seen an intensification of anti-stigma campaigns across Europe seeking to target and ameliorate these harmful consequences. A frequent focus of anti-stigma efforts has been on bringing about a broad scale change in public attitudes towards people experiencing mental illnesses, see for example, Time to Change in the United Kingdom (4, 5) and one of the most effective ways to change public attitudes is to promote social contact between people who experience mental illness and those who do not (6). Social contact allows opportunity for first-hand interactions that reduce the need for reliance on stereotypes of the stigmatised characteristic (7).

Economic conditions can impact on intergroup relations; for example, where there is greater competition for economic resources, there are higher levels of prejudice directed from majority social groups towards minorities such as immigrants (8). Prejudice in these circumstances can arise from both actual and perceived competition for resources (9). Income inequality creates further strain on intergroup relations through mechanisms of status competition (10) and in doing so drives up perceptions of outgroup threat, with damaging effects on social trust and cohesion (11, 12). It is therefore important to consider not only how intergroup relations link with prejudice at the individual level, but also the broader context in which such social interactions take place (13). When economic difficulties intensify, for example during times of recession or economic instability, negative attitudes towards minorities can harden further (14).

People with mental health conditions represent an often-marginalised group who experience entrenched socioeconomic difficulties and for whom the 2007 recession exacerbated existing labour market disadvantage (15, 16). Yet whilst the relationship between socioeconomic conditions and mental illness is well-evidenced, much less is known about whether or not the broader economic context influences public attitudes towards people with mental health conditions.

Historically, much of the focus of stigma research and interventions has been concentrated at the individual level (17) but more recently, attention has turned to the underlying structural factors that produce the societal conditions for stigma to thrive, or indeed that have the potential to mitigate existing prejudices (18). This includes policies, laws, institutional practices and cultural norms which may either intentionally or unintentionally perpetuate stigma.

Measures of stigma in relation to policies, laws and cultural norms have been associated with health inequalities amongst minority groups across a range of indicators such as substance misuse, myocardial infarction and mortality [see Hatzenbuehler and Link (19) for a review]. Specifically relating to mental health stigma, Evans-Lacko et al. (20) used two large European datasets to explore the relationship between public attitudes and self-stigma across fourteen countries. They found that less stigmatising attitudes amongst the general public, higher rates of help seeking, treatment utilisation and better access to mental health information at the country level were associated with lower self-stigma and perceived discrimination at the individual level.

This study seeks to contribute to the evidence base on the structural drivers of stigma associated with mental illness by exploring the relationship between economic conditions and public attitudes. In line with the existing evidence, it focuses specifically on resource competition and inequalities, and uses willingness to speak to a person with a significant mental illness as the outcome variable, a measure of public attitudes and a proxy measure for social distance (20). In doing so, it aims to contribute to further understanding of the role of economic factors in individual experiences of stigma.

A recent global survey suggests that residents in Europe report higher perceptions of social division and tension than in other areas of the world (21) so this research is particularly relevant. Our study uses a cross-national European sample from 2010 to explore the economic context immediately following the Great Recession, to understand what we can learn for this current period of global macroeconomic instability and in the coming years ahead.

## 2. Methods

### 2.1. Sample

The main data source for the analysis, from which the outcome variable and covariates were drawn, was the Eurobarometer, a cross-national survey conducted annually and for 2010, including data on attitudes towards people with mental illness for approximately 26,800 individuals across European countries (22). The survey sample was drawn using multi-stage random probability sampling proportional to population size and density and is representative of the population aged 15 and above in each of the countries [see TNS Opinion and Social (23) for full details of survey methodology]. Interviews were carried out face to face at participant homes, in an appropriate national language (23). The sample for each country includes around 1,000 respondents aside from Luxembourg, Cyprus and Malta where numbers are around 500. This is because sampling is proportionate to population size and so smaller countries have fewer respondents (23). All Eurobarometer data was weighted using the EU27 population weight included in the dataset (22).

The survey used for this analysis represents the second in a special series about mental health across Europe with the first survey taking place in 2006; however, the 2010 Eurobarometer survey is the first and only to explore perceptions of people with mental illness as far as we are aware (22).

### 2.2. Outcome variable

The following Eurobarometer survey question was selected as the outcome variable, in which respondents are asked to choose from one of the following two statements:

• You would find it difficult talking to someone with a significant mental health problem.
• You would have no problem talking to someone with a significant mental health problem.

This measure has been successfully used across a number of studies previously that seek to explore differences in public attitudes towards people experiencing mental health problems across a European sample, and in particular to measure social distance (20, 24).

Answers were recoded into a categorical variable prior to analysis for ease of interpretation. A new variable was derived from the data with 0 = no problem talking to someone with a significant mental health problem and 1 = difficulty talking to someone with a significant mental health problem. “Don't know” responses were excluded from the analysis because it is not possible to interpret this response in relation to desire (or not) for social distance, totalling 9.96% of responses overall (please see Appendix I for a breakdown of responses by country).

### 2.3. Explanatory variables

Two key macro-economic factors were tested for their association with whether or not respondents described difficulty speaking to a person with a significant mental illness. GDP per capita represents the overall financial resources available to a country, whilst income inequality demonstrates how these resources are distributed in the population. Variables were selected from publicly available European data sources and standardised measurements were used to enable cross-national comparison. Data on the 2010 estimates for GDP per capita (Euros) and income inequality (2010 Gini co-efficient) were derived from Eurostat (25).

### 2.4. Covariates

Individual demographic variables were sourced from the same Eurobarometer dataset as the outcome variable. Age and gender were included in the analyses since these factors are known to impact on individual attitudes towards people with mental illness (26). Individual perceptions of financial circumstances (measured as difficulty paying bills over the past 12 months) were also included based on previous research that suggests links between individual and contextual economic factors, and exclusionary attitudes towards people from other minority populations (27).

### 2.5. Analytic strategy

To explore both individual and contextual factors, a random effects logistic regression modelling approach was employed. There is ongoing debate about the acceptable number of contextual levels in multilevel models (28) but in an extensive review of cross-national analyses where multilevel modelling has been used, Bryan and Jenkins (29) recommend that data from upwards of 25 countries should be included to generate reliable estimates, a requirement met by the dataset used here.

All analyses were conducted in Stata version 15.1 (30). Scatter plots were used to explore the relationship between the outcome variable and each of the contextual variables. Individual level variables were added to the model first (model 1), followed by each contextual socioeconomic variable independently (models 2a and b) and a final, full model incorporating all individual and contextual variables (model 3). Likelihood ratio tests confirmed that random effects models were an improved fit compared to ordinary logistic regression once contextual variables were included and intraclass correlations were estimated for each of the random effects models. All effect estimates are reported as odds ratios.

## 3. Results

**Figure 1:**
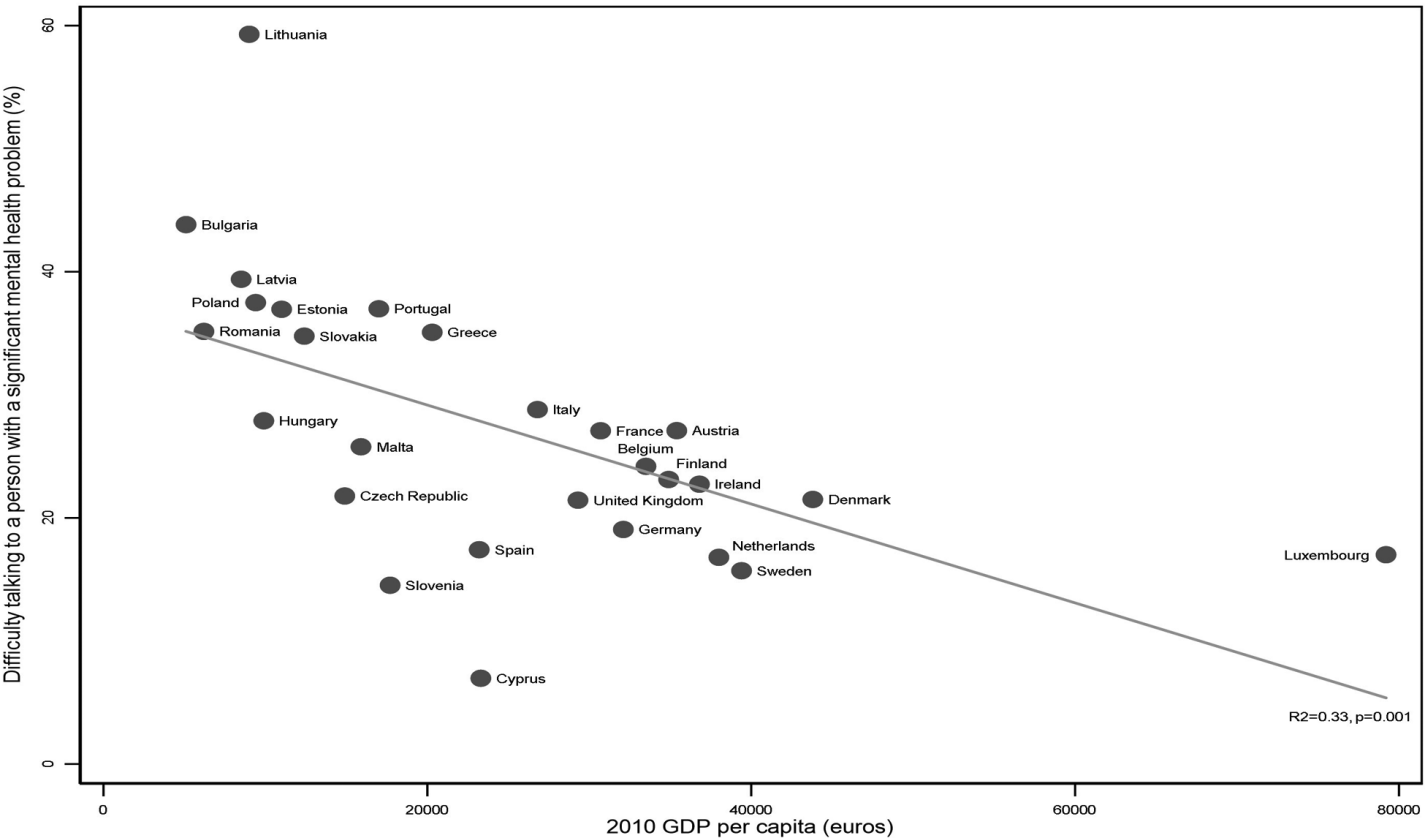
Proportion of respondents finding it difficult to talk to a person with mental illness by GDP per capita across the EU27.

**Figure 2:**
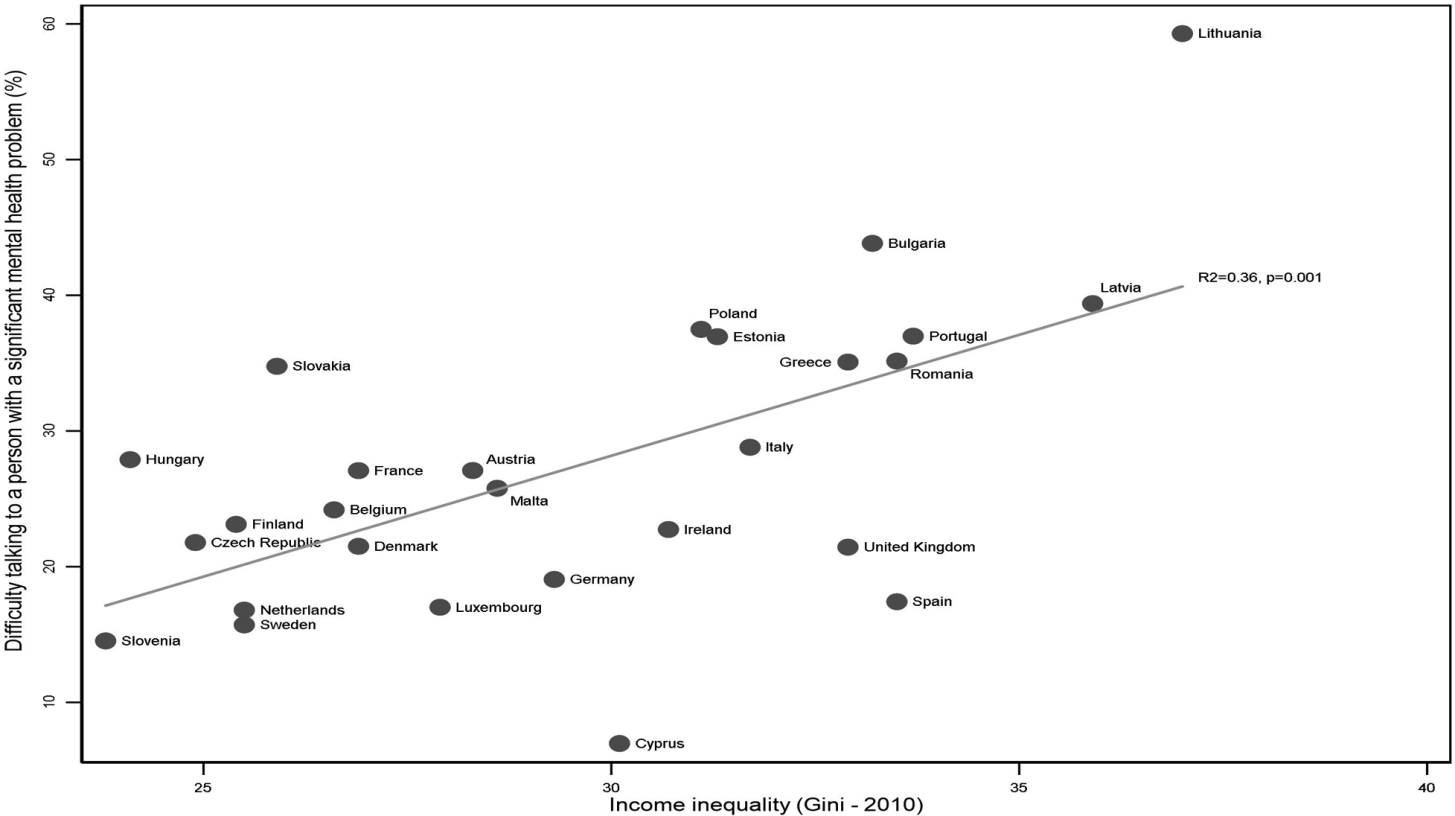
Proportion of respondents finding it difficult to talk to a person with mental illness by income inequality across the EU27.

On average, around a quarter (24.73%) of respondents across the EU-27 reported difficulty speaking to a person with a significant mental illness. Observation of the distribution of responses on the scatter plots (Figures 1, 2) showed that Lithuania (59.59%) and Cyprus (6.97%) were outliers in relation to the proportion of respondents reporting difficulty speaking to a person with a significant mental illness but the decision was taken not to remove these countries because the analysis is intended to be a reflection of the real-world context.

Correlations demonstrated that both income inequality (*R*^2^ = 0.36, *p* = 0.001) and GDP per capita (*R*^2^ = 0.33, *p* = 0.001) were strongly associated with attitudes towards people with mental illness (Figures 1, 2). Higher income inequality and lower GDP per capita were both associated with greater difficulty talking to a person with a significant mental illness.

The main findings are reported in Table 1. Individual financial status was consistently associated with attitudes towards people with mental illness across all models. Compared to those who reported never or almost never having difficulty paying their bills over the past 12 months, those who described difficulties some of the time were 1.25 (1.16, 1.34) times more likely to report having difficulty talking to a person with mental illness and those who described difficulty paying bills most of the time were 1.52 (1.37, 1.68) times more likely.

**Table 1:** Results of logistic regression models exploring associations between individual and contextual economic factors, and difficulty speaking to a person with a significant mental illness across the EU-27.

Variable/model	1	2a	2b	3
	*N* = 23,711	*N* = 23,427	*N* = 23,427	*N* = 23,427
	OR (CI)	OR (CI)	OR (CI)	OR (CI)
Difficulty paying bills never/almost never				
** **From time to time	1.49 (1.40–1.59)***	1.25 (1.17–1.34)***	1.25 (1.17–1.34)***	1.25 (1.16–1.34)***
Most of the time	1.97 (1.79–2.17)***	1.52 (1.37–1.69)***	1.52 (1.37–1.69)***	1.52 (1.37–1.68)***
Age 16–24				
25–39	0.86 (0.78–0.95)**	0.88 (0.80–0.98)*	0.88 (0.80–0.98)*	0.89 (0.80–0.98)*
40–54	0.79 (0.71–0.88)***	0.82 (0.74–0.91)***	0.82 (0.74–0.91)***	0.82 (0.74–0.91)***
55 years and above	0.92 (0.84–1.01)	0.97 (0.88–1.08)	0.97 (0.88–1.08)	0.97 (0.88–1.08)
Male				
Female	0.83 (0.78–0.88)***	0.81 (0.77–0.86)***	0.81 (0.77–0.87)***	0.81 (0.77–0.87)***
GDP per capita (Euros)		0.99 (0.99–0.99)**		0.99 (0.99–0.99)**
Income inequality (Gini)			1.08 (1.03–1.13)**	1.06 (1.01–1.10)**
LR test		603.13***	605.34***	424.23***
ICC		0.05 (0.03–0.09)	0.05 (0.03–0.09)	0.04 (0.02–0.08)

OR, odds ratios; CI, 95% confidence intervals.

* <0.05.

** <0.01.

*** <0.001.

Age was also relevant, with difficulty speaking to a person with a significant mental illness decreasing with age as compared to those in the 16–24 age category, aside from the oldest age group (55 years and above). Gender had a strong and consistent effect, with males more likely to report experiencing difficulty talking to a person with mental illness than female respondents.

Models 2a and b demonstrate that GDP per capita and income inequality were independently associated with the outcome variable and once both contextual socioeconomic variables were included in the same model, the effects for each were maintained.

In the full model (model 3), GDP per capita was associated with difficulty talking to a person experiencing a significant mental illness, so that higher GDP per capita was related to a lower likelihood of reporting difficulty (0.99; 0.99–0.99), albeit this association appears modest. The association between income inequality and the outcome variable is stronger than that of GDP per capita; the more unequal a country, the less likely respondents were to feel comfortable talking to a person with a significant mental illness (1.06; 1.01–1.10).

We estimated odds ratios from the full model at the 20th and 80th percentile of the distribution of each contextual socioeconomic variable, to show the magnitude of effects (data not shown). Respondents from countries at the 80th percentile of the income inequality range were almost two times more likely to report difficulty talking to a person with a significant mental illness compared to the most equal country (Slovenia), whereas those from countries at the 20th percentile of the income inequality range were only 18% more likely to report such difficulty. In terms of GDP per capita, those living in countries at the 20th percentile were 27% less likely, and those at the 80th percentile two and a half times less likely, to report difficulty talking to a person with a significant mental illness compared to the poorest country (Bulgaria).

The comparison between models indicates that the multilevel structure is a better fit for the data as indicated by the likelihood ratio tests (*p* ≤ 0.001). The intraclass correlation associated with each of the models suggests that there is variance present that can be explained by differences between countries as well as within countries (31), here between 4% and 5% depending on the model (see Table 1).

## 4. Discussion

The findings suggest that both individual and contextual economic factors are relevant in determining whether or not people across the EU27 describe difficulty talking to a person with a significant mental illness.

In relation to demographic characteristics, the findings suggest that females are less likely than males to report difficulty talking to a person with a significant mental illness. Age is more complex, with the oldest and youngest respondents in the sample more likely to report difficulty than those in the 25–54 age group. This is at odds with broader findings in social attitudes research, whereby younger respondents often report more tolerant attitudes than those who are older (32), but this finding of a more tolerant middle age bracket has been demonstrated in other mental health attitudinal research from the same time frame. In the Attitudes to Mental Illness survey, a nationally representative study undertaken in the United Kingdom, respondents aged 35–54 gave the most tolerant responses compared to those in both younger and older age brackets, albeit there were nuances by particular questions (33). This suggests that tolerance towards people with a mental illness increases rather than decreases with age, perhaps related to a higher likelihood of having had social contact with a person experiencing mental illness during the life course or possibly because younger adults may be less socially confident in general. It is also potentially a cohort effect although further research would be required to explore this, or it is possible that increasing physical vulnerabilities with advanced age, may increase perceived levels of threat and therefore reduce tolerance compared to middle-aged respondents.

Of all the individual factors, however, it was self-reported financial circumstances that had the strongest association with experiencing difficulty talking to a person with a significant mental illness. Here, there was a gradient of decreasing tolerance for each increment of financial difficulty. Those in the worst financial position were 1.52 (1.37, 1.68) times more likely to report difficulty talking to a person with mental illness than those in the most secure financial position. More recent research from both the UK and China finds a similar association between lower socioeconomic status and a greater desire for social distance from people experiencing mental health problems (34, 35).

The findings also suggest a relationship between public attitudes and the contextual socioeconomic factors included in the models. Higher GDP per capita and lower income inequality were associated with less difficulty talking to a person with mental illness. This means that people with mental illnesses who live in countries with lower GDP per capita and that are more unequal, may be more likely to experience stigma than those who live in countries with different socioeconomic characteristics.

The findings in relation to income inequality are in one way surprising given that there is a higher prevalence of mental illness in more unequal countries (10, 12) which should lead to more opportunities for social contact, thereby reducing overall levels of prejudice. In more unequal countries, however, evidence suggests social contact is less effective as a mechanism for reducing prejudice because intergroup relations are more hierarchical (36). Furthermore, in this context there may be a concentration of individuals experiencing socioeconomic deprivation and mental ill health, which could impact on the attitudes of others in similar socioeconomic positions. Where outgroups are perceived to be larger in size, this has the effect of increasing perceptions of threat (37).

People in countries that are more unequal seem to experience the effects of relative social status more acutely, showing greater status anxiety and an increased desire to enhance self-presentation (38–40). This may lead to greater prejudice and stigmatisation of all groups associated with lower rather than higher social status, including people experiencing mental ill health. Similarly, where GDP per capita is lower, the stress associated with being in a difficult personal financial situation may be more acute because there may be greater competition for economic resources more broadly, though this finding requires further exploration as the effect size here appears to be modest.

### 4.1. Limitations

One of the key limitations of this study is the age of the data because it is possible that there have been changes to attitudes since 2010, although more recent research evidence is mixed (4, 34, 41). Without updated data, it is not possible to know whether the findings are a reflection of this specific time and context; however, the study does provide valuable information on the type of economic circumstances that may be associated with differences in attitudes.

It is also worth noting that whilst the study has focused on those factors that could produce greater intolerance towards people with mental illnesses, across the European countries in the sample, only 24.73% of respondents reported that they would experience difficulty talking to a person with a mental illness. It should be highlighted then, that most people in Europe report having no difficulty talking to a person with a significant mental illness, although there are considerable differences by individual country.

Whilst encouraging, this also raises the issue of social desirability bias. The Eurobarometer is conducted via face to face interview for the majority of respondents (22) and so it is possible that responses have been affected by interviewees wanting to appear more tolerant. Henderson et al. (42) found that whilst questions relating to knowledge about mental illness are not associated with social desirability bias, questions relating to intended behaviour towards people with mental illnesses are, particularly in face to face interviews compared to online surveys. The outcome variable in this study represents an intended behaviour question and therefore social desirability bias could be implicated here. In this case, it is possible that the study findings understate the links between economic factors and attitudes towards people with mental illness.

The outcome variable used here does not directly reference prejudice in the question which may help to address some of the issues around social desirability bias but does produce difficulties in determining the exact meaning of responses. A person may be willing to engage with a person experiencing a significant mental illness, but may, for example, lack confidence, therefore this is not necessarily a measure of desire for social distance. More in-depth, qualitative methodological approaches may be needed to assist in understanding the nuances behind these responses, or alternatively the use of more a more detailed scale to understand differences. This applies also to the “don't know” responses in the sample.

Whilst we do not consider it likely that the association between higher levels of income inequality and increased stigmatization could be fully explained by racial/ethnic heterogeneity, our lack of inclusion of this characteristic could be viewed as a limitation of the analysis. Although racial/ethnic heterogeneity has been suggested as a potential confounder in the past by those sceptical of the impact of inequality on health and wellbeing, research has shown that the association between income inequality and health is independent of racial/ethnic heterogeneity in both international and US state comparisons (43). In addition, as income inequality may itself create the social and economic conditions that increase migration/ethnic heterogeneity, which in turn may be a potential cause of higher levels of downward prejudice and stigmatization, we view ethnic heterogeneity as possibly sitting on the causal pathway (i.e., a mediator, not a confounder).

Within a European context, analytical issues around ethnicity are perhaps even more complicated than in the north American context, where most studies adjust for African-American race and Hispanic ethnicity. In the UK, for example, some south Asian groups have high socioeconomic status and do better in terms of wellbeing measures, while others do worse than the White British average. White Eastern European migrants to the UK and White Roma populations can experience considerable disadvantage, so comparing white to non-white groups obscures important complexity.

The analytical treatment of ethnic heterogeneity in relation to income inequality and outcomes, therefore, is a complex matter, worthy of a further paper and beyond the scope of our current analysis. Further exploration of the role of migration status, ethnicity, language and other measures of identity would be a beneficial next step for future research.

### 4.2. Implications of findings

Both individual demographic differences and contextual economic factors appear to be relevant in explaining the proportion of people who have difficulty talking to a person with a significant mental health problem across European countries. The wider socioeconomic climate is important because it impacts on all people living in a country. Evidence suggests, for example, that income inequality has detrimental effects on health and social outcomes for all residents in more unequal countries (10). Where the wider socioeconomic climate in a country incorporates greater income inequality or lower GDP per capita the population may be more likely to report difficulty speaking to a person with a significant mental illness, though further research is needed to confirm these findings.

Individual perceptions of financial circumstances were consistently and independently associated with difficulty speaking to a person with a significant mental illness which is in keeping with findings relating to attitudes towards minority groups more broadly. It is feasible that the association between individual financial circumstances and attitudes towards mental illness could be a reflection of the wider socioeconomic climate and this would certainly fit with existing research that suggests income inequality is related to more negative outgroup attitudes at the individual level (9, 11, 36). In more unequal countries, the effects of status anxiety are stronger and this impacts on people at all income levels (44). Status anxiety produces more strained interpersonal relationships because it increases concerns about social evaluation, in turn meaning that people are less likely to associate with others who are viewed as a threat to their social status (12). Goffman (45) conceptualises mental illness as a “discredited” characteristic meaning that it is perceived as conferring an inferior social status. It is possible that in socioeconomic climates where status anxiety means that social position carries more weight, people are more likely to disassociate themselves from those perceived as having a discredited characteristic.

This study contributes to the emerging field in mental health research that focuses on the underlying societal level drivers of stigma. These factors are key to improving understanding of the experiences of individuals with mental illnesses since a country may set out intentions for progressive mental health policies in terms of care and treatment, whilst at the same time making economic decisions that perpetuate disadvantage (46) and place strain on intergroup relations. Further research, using more recent data and exploring the underlying mechanisms behind the associations between both micro and macro-economic factors and public attitudes would be beneficial.

Whilst beyond the scope of the current analysis, future research may wish to focus on additional country-level factors that could be associated with GDP per capita and income inequality, and which may impact on attitudes towards people with mental health problems. In particular, differences in healthcare systems, the political, ethnic and religious composition of each country and the prevalence of mental health problems across different populations could each add important detail to the findings. Similarly at the individual level, additional context in relation to individual demographic variables may further strengthen the analyses, for example, ethnicity, religion and educational level. Although not possible with this particular dataset, it would also be interesting to know whether the perceived socioeconomic status of the person experiencing mental illness had any impact on public attitudes.

Significant social, economic and political change seems likely in the United Kingdom and potentially across Europe in the coming years and this may potentially impact on people with mental health conditions, producing further social exclusion. Reducing existing stigma, whilst ensuring that people with mental health conditions do not become further marginalised in this context, should be key priorities for mental health practitioners and policy makers alike. Targeting income inequality should be the first step to improving intergroup relations, and therefore potentially attitudes towards people experiencing mental illness, ultimately reducing the harmful effects of stigma.

## Data Availability

The original contributions presented in the study are included in the article/**Supplementary Material**, further inquiries can be directed to the corresponding author.
