# Supplementary material for: The socioeconomic context of stigma: examining the relationship between economic conditions and attitudes towards people with mental illness across European countries
Source: Front Epidemiol. 2023 Jul 19;3:1076188. doi: 10.3389/fepid.2023.1076188 (PMC10910911; doi:10.3389/fepid.2023.1076188)
Supplement: Supplementary file 1 [file Table1.docx]

Appendix I: Response data for outcome variable

| **Country** | **Difficulty talking to someone with a significant mental health problem (%)** | **No problem talking to someone with a significant mental health problem (%)** | **Don’t know (%)** | **Total** |
| --- | --- | --- | --- | --- |
| France | 270 (25.64) | 727 (69.04) | 56 (5.32) | 1,053 |
| Belgium | 240 (23.26) | 752 (72.87) | 40 (3.88) | 1,032 |
| The Netherlands | 166 (16.44) | 822 (81.39) | 22 (2.18) | 1,010 |
| Germany | 263 (16.71) | 1117 (71.01) | 193 (12.26) | 1,573 |
| Italy | 252 (24.14) | 623 (59.67) | 169 (16.19) | 1,044 |
| Luxembourg | 83 (16.44) | 405 (80.20) | 17 (3.37) | 505 |
| Denmark | 207 (20.62) | 756 (75.30) | 41 (4.08) | 1,004 |
| Ireland | 201 (19.82) | 683 (67.36) | 130 (12.82) | 1,014 |
| Great Britain | 208 (20.43) | 762 (74.85) | 48 (4.72) | 1,018 |
| Greece | 328 (32.80) | 607 (60.70) | 65 (6.50) | 1,000 |
| Spain | 162 (16.10) | 768 (76.34) | 76 (7.55) | 1,006 |
| Portugal | 334 (32.36) | 569 (55.14) | 129 (12.50) | 1,032 |
| Finland | 206 (20.50) | 685 (68.16) | 114 (11.34) | 1,005 |
| Sweden | 154 (15.34) | 826 (82.27) | 24 (2.39) | 1,004 |
| Austria | 237 (23.49) | 638 (63.23) | 134 (13.28) | 1,009 |
| Cyprus | 32 (6.34) | 427 (84.55) | 46 (9.11) | 505 |
| Czech Republic | 206 (20.16) | 740 (72.41) | 76 (7.44) | 1,022 |
| Estonia | 344 (34.40) | 587 (58.70) | 69 (6.90) | 1,000 |
| Hungary | 258 (24.81) | 667 (64.13) | 115 (11.06) | 1,040 |
| Latvia | 373 (37.00) | 574 (56.94) | 61 (6.05) | 1,008 |
| Lithuania | 533 (52.46) | 366 (36.02) | 117 (11.52) | 1,016 |
| Malta | 107 (21.40) | 308 (61.60) | 85 (17.00) | 500 |
| Poland | 328 (32.80) | 547 (54.70) | 125 (12.50) | 1,000 |
| Slovakia | 339 (32.85) | 636 (61.63) | 57 (5.52) | 1,032 |
| Slovenia | 136 (13.53) | 800 (79.60) | 69 (6.87) | 1,005 |
| Bulgaria | 359 (35.72) | 460 (45.77) | 186 (18.51) | 1,005 |
| Romania | 232 (22.01) | 428 (40.61) | 394 (37.38) | 1,054 |
| **Total** | **6,629 (24.73)** | **17,500 (65.29)** | **2,671 (9.96)** | **26,800** |
